# Latent Psychotic Symptom Profiles Amongst People Who Use Methamphetamine: What Do They Tell Us About Existing Diagnostic Categories?

**DOI:** 10.3389/fpsyt.2018.00578

**Published:** 2018-11-19

**Authors:** Rebecca McKetin, Alexandra Voce, Richard Burns, Robert Ali, Dan I. Lubman, Amanda L. Baker, David J. Castle

**Affiliations:** ^1^Faculty of Health Sciences, National Drug Research Institute, Curtin University, Perth, WA, Australia; ^2^National Drug and Alcohol Research Centre, University of New South Wales, Sydney, NSW, Australia; ^3^Research School of Population Health, Australian National University, Canberra, ACT, Australia; ^4^School of Medical Sciences, University of Adelaide, Adelaide, SA, Australia; ^5^Turning Point, Eastern Health and Eastern Health Clinical School, Monash University, Melbourne, VIC, Australia; ^6^School of Medicine and Public Health, Faculty of Health, University of Newcastle, Callaghan, NSW, Australia; ^7^St Vincent's Hospital, Fitzroy, VIC, Australia; ^8^Department of Psychiatry, University of Melbourne, Melbourne, VIC, Australia

**Keywords:** methamphetamine, amphetamine-related disorders, psychotic disorders, schizophrenia, diagnosis, psychosis

## Abstract

The inability to distinguish clearly between methamphetamine-related psychosis and schizophrenia has led to the suggestion that “methamphetamine psychosis” does not represent a distinct diagnostic entity but rather that the drug has triggered a vulnerability to schizophrenia. We tested this possibility by exploring the latent class structure of psychotic symptoms amongst people who use the drug and examining how these latent symptom profiles correspond to a diagnosis of schizophrenia. Latent class analysis was carried out on the lifetime psychotic symptoms of 554 current methamphetamine users, of whom 40 met the DSM-IV criteria for schizophrenia. Lifetime diagnoses of schizophrenia and individual psychotic symptoms were assessed using the Composite International Diagnostic Interview. The chosen model found 22% of participants had a high propensity to experience a wide range of psychotic symptoms (schizophrenia-like), whereas the majority (56%) more specifically experienced persecutory delusions and hallucinations (paranoid psychosis) and had a lower probability of these symptoms than the schizophrenia-like class. A third class (22%) had a low probability of all symptoms, with the exception of 34% reporting persecutory delusions. Participants in the schizophrenia-like class were more likely to meet diagnostic criteria for schizophrenia (26 vs. 3 and 1% for each of the other classes, *p* < 0.001) but the diagnosis failed to encompass 74% of this group. These results are consistent with there being a distinction between schizophrenia and methamphetamine-related psychotic symptoms, both in terms of the propensity to experience psychotic symptoms, as well as the symptom profile; however, this distinction may not be captured well by existing diagnostic classifications.

## Introduction

Both current international classification systems for mental disorders [i.e., the International Statistical Classification of Diseases and Related Health Problems (ICD), 10th Revision (1) and the Diagnostic and Statistical Manual, 5th edition, DSM-5 (2)] differentiate between psychosis related to methamphetamine use (under the diagnosis of a substance-induced psychosis) and primary psychotic disorders, such as schizophrenia. However, the commonality between the symptom profile of methamphetamine psychosis and acute paranoid schizophrenia (3, 4) often makes it difficult to make a clear diagnosis, particularly in the early stages of psychosis when prognostic information is not yet available (5, 6). This frustration has led to concerns about the clinical utility of the diagnostic categories and the potential ramifications of misdiagnosis and failure to intervene early, particularly as substance-induced presentations are often used as a justification to exclude individuals from psychiatric care (7).

Most of the previous research that has attempted to differentiate between schizophrenia and methamphetamine-related psychosis use has done so by comparing symptom profiles cross-sectionally. For example, Medhus and colleagues compared individuals presenting with psychosis by whether or not they tested positive for methamphetamine psychosis, and found no significant differences in the severity of positive symptoms (5). Hides et al. (6) similarly failed to find differences in the severity of overall positive or negative symptoms in methamphetamine users who met DSM-IV diagnostic criteria for substance-induced psychosis and those who met DSM-IV criteria for a primary psychotic disorder. Srisurapanont et al. (8) examined more specific symptoms in methamphetamine psychosis, and, using cluster analytic techniques, found evidence of negative, positive and affective symptom clusters, which were almost the same as that seen in a comparison group of patients diagnosed with schizophrenia.

This lack of a clear diagnostic boundary between methamphetamine-related psychosis and schizophrenia has led Bramness and colleagues to propose that methamphetamine psychosis does not represent a unique diagnostic entity, but would be better conceptualized as a triggering of a schizophrenia spectrum disorder in vulnerable individuals (9). This theory is couched in the stress-vulnerability framework, whereby vulnerability to psychosis occurs along a continuum of risk, and exposure to methamphetamine interacts with this latent risk to precipitate psychosis. Conceptualized within this framework, a psychosis precipitated by methamphetamine need not be considered a separate diagnostic entity from a primary psychotic disorder. This possibility opens the door for such individuals to be provided with an early intervention approach for psychosis, including antipsychotic treatment, as would be the case for individuals with a primary psychotic disorder.

On the other hand, methamphetamine use is associated with different prognostic outcomes amongst people presenting with psychosis (10–12). Case reports (13) and experimental inductions of methamphetamine psychosis (4) suggest that it is a transient phenomenon that does not warrant ongoing anti-psychotic treatment. There is also emerging evidence that the symptom profile associated with methamphetamine use can be distinguished from that associated with primary psychotic disorders, in that specific types of psychotic symptoms (particularly non-persecutory delusions) are risk markers for more persistent psychosis (14) and a diagnosis of a primary psychotic disorder (15, 16). Together, this evidence suggests a potential clinical benefit in identifying methamphetamine-related psychosis as distinct from non-organic psychotic processes.

One way to test whether there is any merit in retaining the diagnostic category of methamphetamine-induced psychosis (cf. the triggering of schizophrenia) is using latent class analysis. Latent class analysis classifies population heterogeneity into categorical groups of homogeneous individuals which may have implications for classification (i.e., diagnosis), prognosis (i.e., longitudinal course), and treatment (i.e., propensity to respond to different treatments) (17). Latent class analysis has been increasingly applied in psychiatry to identify subgroups of patients or clinical markers that may have clinical utility but which are obscured by more traditional methods of analysis, such as pairwise group comparisons that presuppose diagnostic structures and their relationship to clinical characteristics (18–20). In latent class analysis, the presence of a group of people who are homogenous in their symptom profile should present as a single latent class. The presence of more than one latent class would suggest multiple groups of individuals who are distinguishable based on their symptom profile, and would be consistent with a need for different diagnostic categories to reflect these different symptom typologies.

Here we use latent class analysis to examine whether there is evidence of different classes of psychosis amongst people who use methamphetamine, as well as to understand how these classes correspond to the diagnostic category of schizophrenia. We hypothesized that if psychosis amongst people who use methamphetamine reflects the triggering of schizophrenia, then we would detect a single latent profile of psychotic symptoms, thus reflecting the symptom profile associated with schizophrenia. Conversely, if methamphetamine induced a psychosis that was distinct from schizophrenia then this should manifest as a separate class reflecting the symptom profile associated with methamphetamine-induced psychosis. Essentially, the presence of two or more latent classes would suggest different psychosis typologies in the population, and this would be more consistent with a need for multiple diagnostic categories to capture the heterogeneity in psychosis amongst people who use methamphetamine.

## Materials and method

### Participants and procedure

Participants were drawn from two Australian-based studies of methamphetamine users (21, 22). Data on 178 participants were taken from a cross-sectional survey conducted in Canberra in 2016–17 of volunteers recruited from the general community (via advertisements at needle and syringe programs, online and other public locations, and word of mouth) who used methamphetamine at least monthly and who were aged 18 years or older (21). Data for a further 376 participants were drawn from a longitudinal cohort study, the Methamphetamine Treatment Evaluation Study (MATES) (22, 23), conducted in Sydney and Brisbane from 2006 to 2011, and which included 400 participants seeking treatment for methamphetamine use; and, a further 101 dependent methamphetamine users recruited from the community. MATES participants had to be 16 years or older and not have been incarcerated, in drug treatment or any in-patient treatment for the month prior to enrollment. Participants from the MATES cohort were not included if they did not meet DSM-IV criteria for methamphetamine dependence in the year prior to recruitment (*n* = 17) or they did not complete the 3-month follow-up interview where a diagnosis of schizophrenia was made (*n* = 92). Interviews were conducted face-to-face or by phone. All participants were volunteers who provided either written or verbal informed consent and were reimbursed (up to AUD40 per interview). Verbal informed consent procedures were approved by the institutional Human Research Ethics Committee.

### Measures

#### Psychosis measures

A DSM-IV lifetime diagnosis of schizophrenia was made using the Composite International Diagnostic Interview (CIDI) Version 2.1 (24). Negative symptoms, disorganization and catatonia were not assessed because of the difficulty assessing these symptoms retrospectively across the participant's lifespan based on current self-report. Lifetime psychotic symptoms were based on the symptom criteria for schizophrenia as assessed in the pertinent section of the CIDI (24). Delusions were grouped as persecutory, thought projection, thought interference, passivity, reference, other delusions (erotomania, jealousy, mind reading). Hallucinations were categorized as complex auditory hallucinations, other auditory hallucinations, visual hallucinations, and other hallucinations (olfactory, gustatory and tactile). See the Supplementary material for further detail.

#### Substance use

Days of methamphetamine use and other substance use (cannabis, heroin, cocaine, ecstasy, hallucinogens, alcohol, and tobacco) in the previous 4 weeks was assessed using the Opiate Treatment Index (25). Self-reported abstinence from methamphetamine was confirmed in a sub-sample of the MATES cohort using hair toxicology, with false reporting of abstinence occurring in only 6% of cases (22). Other methamphetamine use measures included age of first use, main route of methamphetamine administration in the previous month, as well as severity of methamphetamine dependence in the previous month, assessed using the Severity of Dependence Scale (SDS) (26). Dependence on methamphetamine was defined as a score of 4 or greater on the SDS scale, which corresponds to a CIDI diagnosis of severe dependence with 71% sensitivity and 77% specificity (27). Baseline data are reported for the MATES participants.

### Statistical analysis

Latent class analysis in MPlus version 7.2 (28) was applied to the binary symptom variables using a maximum likelihood estimator with robust standard errors. Latent models were fitted using 600 random starting values to ensure replication of the final log-likelihood value. Modeling was performed sequentially by examining whether each additional class significantly improved the fit of the model to the data, as indicated by the Voung-Lo-Mendell-Rubin Likelihood Ratio Test (VLMR LRT) and the parametric bootstrapped Likelihood Ratio Test (BLRT) (29, 30). Owing to a sample size that may be sensitive to small chi-square changes, entropy, the Bayesian Information Criterion (BIC) and Akaike Information Criterion (AIC) were also used to compare model fit. Consideration of all goodness of fit indices and parsimony determined the number of classes to be extracted. Participants' most likely class was determined from the latent class posterior distribution.

Other data analyses were performed using Stata SE version 14.1 (31). Group comparisons were made using a Pearson's Chi Square test for categorical data, *t*-tests for continuous data, and a median comparison test for skewed continuous data (where medians and interquartile ranges [IQR] are reported rather than means and standard deviations [SDs]). Receiver Operating Characteristics was conducted using the “roctab” command and the “pvenn2” was used to produce the related Venn diagram. All tests were two-sided with significance set at *p* < 0.05.

## Results

### Characteristics of the sample

Participants (*n* = 554) had a mean (SD) age of 34.3 (9.5) years, 70% were male, and 89% were Australian born. They had used methamphetamine for a mean (SD) of 15.4 (9.0) years and they had used on a median (IQR) of 14 (6–20) days in the previous month. For participants who had used methamphetamine in the previous month (95%), 86% usually took crystalline methamphetamine. The main route of administration was injection (75%), with 18% smoking and 6% swallowing or snorting the drug. The most commonly used other drugs in the previous month were tobacco (95%), cannabis (79%), and alcohol (68%), with other drugs being used less commonly (heroin 31%, ecstasy 21%, cocaine 21%, inhalants 6%, and hallucinogens 6%).

Lifetime psychotic symptoms were reported by 87% of participants, most commonly persecutory delusions (74%), auditory hallucinations (49%: 27% complex and 23% other), visual hallucinations (43%) and other hallucinations (56%) (Table 1). Seven percent of participants (*n* = 40) met the DSM-IV criteria for a lifetime diagnosis of schizophrenia.

**Table 1:** Participant characteristics by latent class.

	**“Schizophrenia-like” (*n* = 123)**	**“Paranoid psychosis” (*n* = 309)**	**“Few symptoms” (*n* = 122)**	**Total sample (*n* = 554)**
**SYMPTOMS (%)**				
Persecutory delusions	98	80^***^	34^***^^†††^	74
Delusions of reference	54	10^***^	0^***^^†††^	18
Thought projection	74	12^***^	1^***^^†††^	23
Thought interference	63	4^***^	0^***^^†††^	16
Delusions of passivity	58	5^***^	1^***^^†††^	16
Other delusions	75	27^***^	2^***^^†††^	32
Visual hallucinations	70	49^***^	0^***^^†††^	43
Complex auditory hallucinations	74	17^***^	3^***^^†††^	27
Other auditory hallucinations	11	36^***^	0^***^^†††^	23
Other hallucinations^a^	85	67^***^	0^***^^†††^	56
**DEMOGRAPHICS**				
Age (median years)	33	33	35	34
Male (%)	71	69	75	71
Years of schooling (median)	10	10	10	10
Unemployed (%)	82	75	73	76
Immigrant (%)	17	8^**^	12	11
**METHAMPHETAMINE USE**				
Duration of use (median years)	15	14	15	14
Days of use (median)	14	15	13	14
Injecting (%)	80	71	71	73
SDS score (%)	9	8	7	8
Dependent (%)	81	79	74	78
**OTHER DRUGS USED IN THE PAST MONTH (%)**				
Tobacco	98	93^*^	96	95
Cannabis	82	79	78	79
Alcohol	72	69	61	68
Ecstasy	15	25^*^	14^†^	21
Cocaine	20	23	20	21
Hallucinogens	6	7	6	6
Inhalants	6	7	2	6
Heroin	28	33	30	31
No. other drug classes used in past month (mean)	3.3	3.4	3.1^†^	3
DSM-IV criteria for schizophrenia (%)	26	3^***^	0^***^	7

* *p < 0.05*,

** *p < 0.01*,

*** *p < 0.001, relative to the schizophrenia-like class*.

† *p < 0.05*,

††† *p < 0.001, relative to the paranoid psychosis class*.

a *Tactile, gustatory or olfactory*.

### Latent class analysis

A two-class model significantly improved fit over a one class model (Table 2). A three-class model further improved model fit. The three-class model was selected based on significant VLMR and LRT tests, and lower AIC and adjusted BIC (Table 2). A sensitivity analysis was conducted that included a covariate in the analysis that identified the study from which participants were recruited to confirm that this was not unduly influencing the classes detected. A comparable pattern of results was found. Analysis of a four-class model is not reported as the highest log-likelihood value was not replicated and thus was excluded from further consideration.

**Table 2:** Model fit statistics for latent class analysis.

	**Full sample (*****N*** = **554)**	
	**Two-class**	**Three-class**
Class membership	1. *n* = 169 (31%) 2. *n* = 385 (69%)	1. *n* = 123 (22%) 2. *n* = 309 (56%) 3. *n* = 122 (22%)
Bootstrap LRT (*p*-value)	−7475 (< 0.001)	−7119 (< 0.001)
Entropy	0.796	0.761
AIC/Adjusted BIC	5498/5522	5384/5421
VLMR LRT (*p*-value)	−7475 (< 0.001)	−7119 (0.003)

*Akaike's information criterion (AIC), Bayesian information criterion (BIC), Voung-Lo-Mendell-Rubin (VLMR), likelihood ratio test (LRT)*.

**Figure 1:**
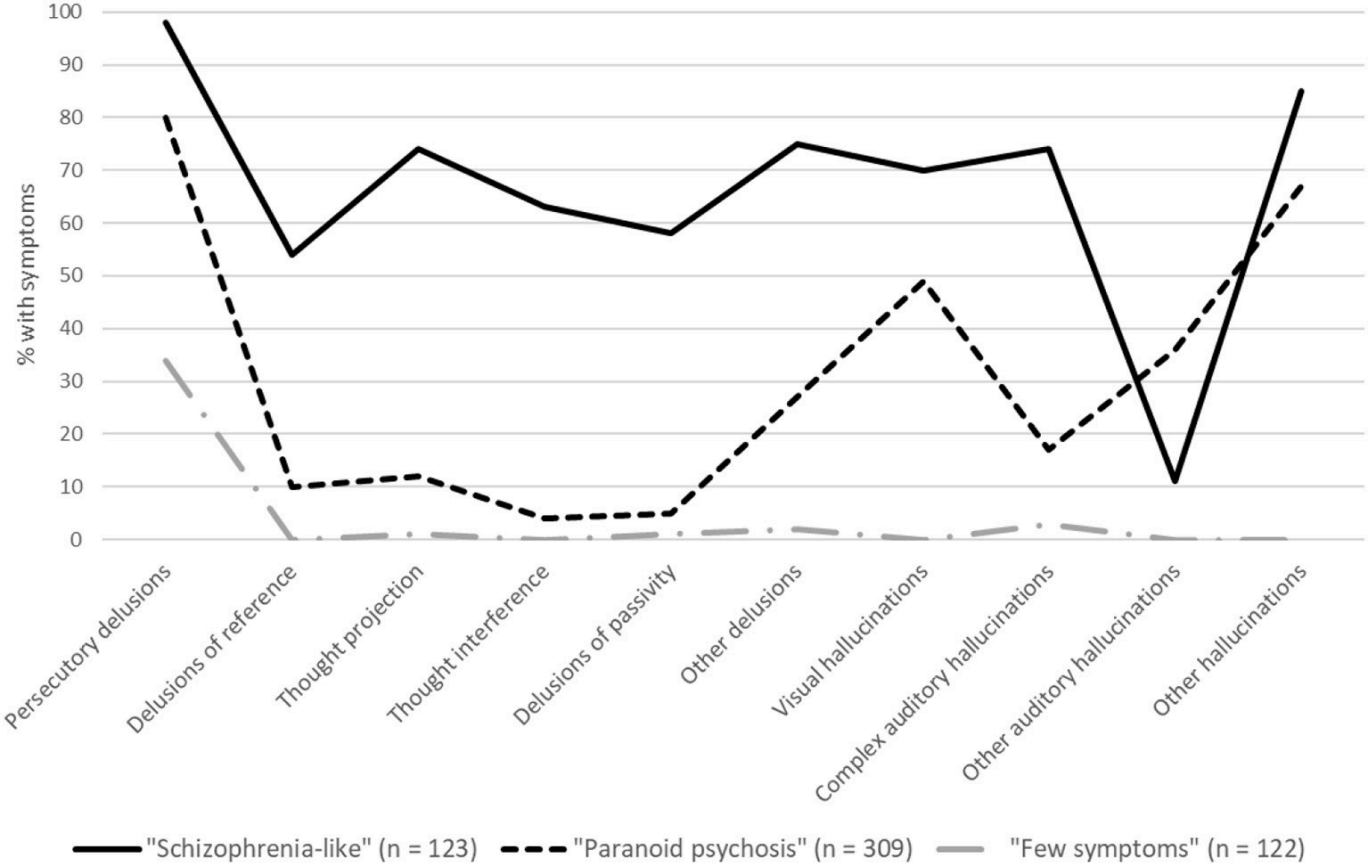
Lifetime symptom prevalence for the three-class model.

The symptom profile associated with each class in the three-class model is shown in Figure 1, and the characteristics of each class are shown in Table 1. The first class (22% of participants) had a very high probability of reporting almost all types of psychotic symptoms and were significantly more likely to meet the DSM-IV criteria for schizophrenia than the other two latent classes (26 vs. 3 and 0%, respectively; Table 1). We labeled this group “schizophrenia-like.” The majority of participants (56%) had a symptom profile that was characterized more specifically by persecutory delusions (80%) and various hallucinations (17–67%; labeled “paranoid psychosis”). Participants in this class and had a significantly lower probability of all symptom types than the schizophrenia-like group and only 3% met the DSM-IV diagnostic criteria for schizophrenia. The third class comprised a minority of participants (22%) who had a very low probability of all symptoms with the exception of 34% reporting persecutory delusions (labeled “few symptoms”); none of the participants in this class met the DSM-IV diagnostic criteria for schizophrenia.

There were few differences in the demographic or polysubstance use characteristics of the three classes. The paranoid psychosis class was less likely to be immigrant, more likely to use ecstasy and less likely to smoke tobacco, while the few symptom class had lower levels of polysubstance use (Table 1).

### Receiver operating characteristics (ROC) analysis

**Figure 2:**
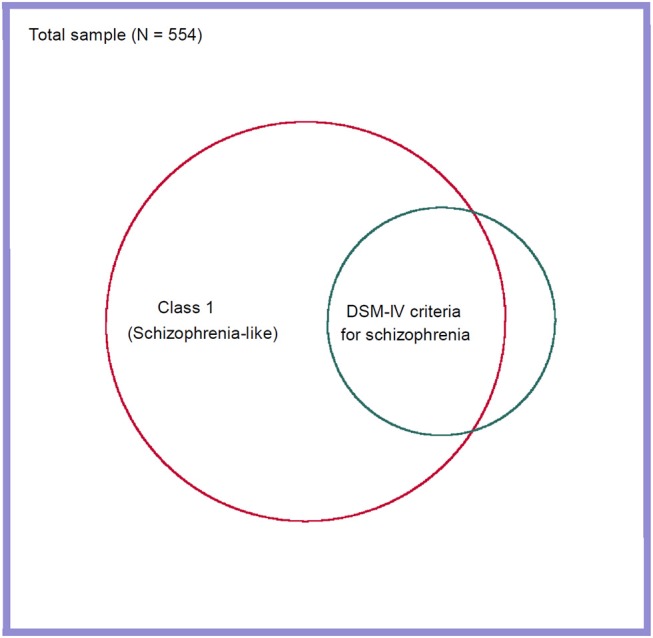
Venn diagram showing overlap between the schizophrenia-like class and a DSM-IV diagnosis of schizophrenia.

To assess concordance between the DSM-IV diagnosis of schizophrenia and the schizophrenia-like class we detected in our LCA analysis, we conducted a ROC analysis with the schizophrenia-like class (*n* = 123) as the reference variable and the DSM-IV diagnosis of schizophrenia (*n* = 40) as the class variable. Although there was significant concordance between meeting the DSM-IV criteria for schizophrenia and membership in the schizophrenia-like class (ROC area = 0.62, 95% CI 0.58–0.66), and the DSM-IV criteria had good specificity in detecting participants in the schizophrenia-like class (98%), sensitivity was poor (26%). Thus, 74% of participants who fell into the schizophrenia-like class did not meet the DSM-IV diagnostic criteria for schizophrenia (Figure 2).

## Discussion

These findings suggest the presence of three latent classes of psychosis vulnerability amongst people who use methamphetamine, including two distinct classes of individuals who have vulnerability to psychotic symptoms, but differ in both their symptom profile and their probability of psychotic symptoms. The identification of multiple relatively distinct latent classes of psychosis (i.e., different typologies of psychosis) in this population is inconsistent with the notion of a single diagnostic category, as would be expected if methamphetamine was triggering schizophrenia. Importantly, we found latent symptom profiles that aligned conceptually with the existing diagnostic groupings of schizophrenia and methamphetamine-induced psychosis. Specifically, the larger of the two groups had a symptom profile comprised of persecutory delusions and hallucinations, consistent with the classic notions of methamphetamine-induced psychosis (32). In contrast, a minority of people who used the drug had a comparatively high probability of all psychotic symptoms and were significantly more likely to meet the DSM-IV diagnostic criteria for schizophrenia.

The presence of multiple latent psychotic symptom profiles in this population does not preclude a common etiology for psychosis vulnerability, in the sense that different psychotic symptom profiles could plausibly stem from a common underlying vulnerability, as suggested by Bramness et al. (9). For example, influenza results in symptom clusters in multiple organ systems, which present at different time in the course of the illness, and can vary in how they are expressed between individuals. However, the reliance on clinical syndromes over etiological mechanisms to define psychiatric disorders means that diagnostic categories need to carry weight in their usefulness to describe and treat patients, and to understand their likely prognosis. In this sense, our data suggest that there are meaningfully different sub-populations of psychosis amongst people who use methamphetamine (both in terms of their propensity to experience psychotic symptoms and their symptom profile). Further research is needed to determine the prognostic utility of these identified typologies and how they could be better captured using diagnostic criteria.

Although the identified classes of psychosis vulnerability bear some resemblance to existing diagnostic categories, individuals who had a high probability of psychosis (the schizophrenia-like class) were not sufficiently well captured by the diagnostic criteria for schizophrenia, or at least not as they are assessed through the CIDI. This lack of sensitivity to identify methamphetamine users who have a high probability of psychosis is likely to underpin the challenges faced by clinicians in being able to apply diagnostic criteria to identify individuals who would benefit from early intervention strategies for a psychotic disorder (7). That said, we also show that the majority of individuals who use methamphetamine do not reach a threshold of symptom severity associated with schizophrenia, and this has potentially important implications for treatment, in that these individuals may not benefit from sustained antipsychotic treatment and may require a different model of care.

The poor sensitivity of the diagnostic criteria for schizophrenia to detect individuals who we identified as having a schizophrenia-like psychosis is likely to be due to the poor sensitivity of the CIDI to detect schizophrenia (relative to clinician ratings) more generally (33). However, it could also reflect poor sensitivity of the DSM criteria in the context of comorbid methamphetamine use. The latter view has been flagged by researchers and clinicians (7), and suggested by prospective data, in which 30% of individuals initially diagnosed with an amphetamine-related psychosis are re-diagnosed with a primary psychotic disorder within 10 years (34). The existence of this orphan category of individuals who had a high probability of psychosis, but who failed to meet the DSM-IV criteria for schizophrenia, may explain clinical descriptions of a persistent or prolonged form of psychosis amongst people who use methamphetamine (as distinct from schizophrenia), and that this phenomena shares commonality with schizophrenia both in terms of its familial morbidity (35) and symptom profile (14). These individuals may have an underlying vulnerability to schizophrenia that is triggered by methamphetamine, or conversely, are using the drug as a form of self-medication to manage a premorbid vulnerability or prodromal state.

Our finding suggest that assessing the past experience of specific types of psychotic symptoms may help to identify methamphetamine-related psychosis patients who would benefit from an early intervention for a psychotic disorder. Specifically, we found a broader symptom profile (i.e., presence of non-persecutory delusions and complex auditory hallucinations) amongst individuals who had a schizophrenia-like psychosis profile. This finding aligns with our previous research which found that the presence of these symptoms was associated with psychosis that persisted beyond methamphetamine use and that their presence in first episode psychosis portended a subsequent diagnosis of schizophrenia (14–16). Conversely, evidence of a second class of people with paranoid psychosis, similar to the current conceptualization of methamphetamine-induced psychosis, suggests that individuals who report having only ever experienced persecutory delusions (with or without hallucinations) are likely to be at much lower risk of having schizophrenia and may benefit more from substance use treatment to reduce their risk of subsequent psychotic episodes rather than ongoing antipsychotic treatment. A caution here is that we did not have prognostic data available to validate the clinical utility of the latent classes of psychosis that we detected. For this reason we suggest that the monitoring of symptoms, including their response to clinical interventions, remains imperative.

The presence of a sub-group of individuals who report having experienced few psychotic symptoms, or only paranoia, suggests differential vulnerability to methamphetamine-related psychosis. This observation is consistent with previous literature showing that not all people who use the drug develop psychotic symptoms (36) and the continuum of psychosis vulnerability observed at a population level, this owing to the many genetic and environmental factors thought to contribute to psychosis risk (37). The preponderance of persecutory delusions across all classes of psychosis vulnerability, even amongst participant with no other psychotic symptoms, may reflect suspiciousness related to the illicit-drug using context (e.g., fear of retribution, social conflict, police surveillance). It could also reflect a continuum of vulnerability whereby persecutory delusions are expressed at lower levels of psychosis proneness, and conversely, that higher levels of vulnerability are required for the expression of hallucinations and non-persecutory delusions (36).

A limitation of the current study was that schizophrenia was the only primary psychotic disorder diagnosed and only a small number of participants met the criteria for this disorder. However, we did not exclude psychotic symptoms that occurred in the context of depression or mania when making this diagnosis, and therefore any participants meeting the symptom criteria for positive symptoms (i.e., presence of delusions and/or hallucinations) in the context of these affective disturbances would have been captured under a diagnosis of schizophrenia. In addition, we used a DSM-IV diagnosis of schizophrenia (cf. DSM-5) and we did not include negative symptoms, or symptoms of disorganization or catatonia when making the diagnosis. The inclusion of these symptoms may have improved alignment between the diagnosis of schizophrenia and our high probability of psychosis sub-population, as some of these symptoms have been shown to differentiate between sub-classes of psychosis proneness and predict conversion to psychotic disorders (20). Finally, we did not attempt to diagnose substance-induced psychosis in this sample, so although we observed a symptom profile consistent with methamphetamine-related psychosis, we cannot confirm whether these participants would have met criteria for this disorder.

The finding that immigrants had a higher probability of psychosis is consistent with migration being a risk factor for psychosis (38), but it may also reflect racial differences in psychosis risk. It is important to note that our sample consisted of mostly Australian born individuals, and this may affect how the findings generalize to other racial groups and cultures. There was also a high rate of polysubstance use, including cannabis use, in this sample. Cannabis use in particular has been related to increased risk of developing a psychotic disorder (39, 40), and more cannabis frequent use is associated with an elevated occurrence of psychotic symptoms amongst people who use methamphetamine (23). However, we did not find any evidence of greater polysubstance use, including cannabis use, amongst participants who had a high probability of psychosis in this sample.

In sum, we demonstrate the importance of retaining the diagnostic category of methamphetamine-related psychosis (i.e., substance-induced psychosis in the DSM-5) as an alternative to schizophrenia, as it is clear that the majority of people who use methamphetamine have a relatively low propensity to experience psychotic symptoms that would meet the criteria for schizophrenia, and have a different symptom typology. Prognostic data would be needed to confirm the full diagnostic utility of these latent sub-populations of psychosis vulnerability. However, the cross-sectional perspective provided here cautions against assuming that all psychosis arising in the context of methamphetamine use reflects schizophrenia. In addition, we found that the DSM-IV diagnostic criteria for schizophrenia had limited utility for identifying methamphetamine users who had a high probability of psychosis, suggesting that further development of the criteria are needed to improve the detection of psychotic disorders in this population.

## Ethics statement

The research was approved by the Australian National Human Research Ethics Committee (2015/638). All participants provided verbal informed consent prior to participation. Verbal consent rather than written consent was obtained in order to protect the confidentiality of participants.

## Author contributions

RM was the lead investigator on the two studies from which the data were drawn; she conceived of and drafted the manuscript. AV collected survey data and conducted the latent class analysis. RB, DC, and RM provided Ph.D. supervision to AV in undertaking these activities. AB, DL, and RA were investigators on the MATES cohort. All authors have contributed to the drafting of the final manuscript.

### Conflict of interest statement

DL has provided consultancy advice to Lundbeck and Indivior, and has received travel support and speaker honoraria from Astra Zeneca, Indivior, Janssen, Lundbeck, Servier, and Shire. The remaining authors declare that the research was conducted in the absence of any commercial or financial relationships that could be construed as a potential conflict of interest.
