# Supplementary material for: Latent Psychotic Symptom Profiles Amongst People Who Use Methamphetamine: What Do They Tell Us About Existing Diagnostic Categories?
Source: Front Psychiatry. 2018 Nov 19;9:578. doi: 10.3389/fpsyt.2018.00578 (PMC6262399; doi:10.3389/fpsyt.2018.00578)
Supplement: Supplementary file 1 [file Data_Sheet_1.docx]

**Supplementary Material**

**CIDI symptoms groupings:** Delusions were grouped as persecutory (beliefs about being spied on, talked about or laughed at, followed or plotted against, or secretly tested), thought projection (hearing other people’s thoughts; others hearing their thoughts), thought interference (convinced strange thoughts were being put directly into their mind, or someone could steal their thoughts), passivity (convinced they were under control of a power or force, or felt strange forces working on them, e.g. x-rays or laser beams), reference (believed that they were being sent special messages through television/radio, or a book, newspaper or song was meant only for them), other delusions (erotomania, jealousy, mind reading). Hallucinations were categorized as complex auditory hallucinations (voices commenting on the participant’s behavior or discussing the participant; two or more voices talking to each other; the participant having a two-way conversation with voices, voices coming from the participant’s body), other auditory hallucinations, visual hallucinations, and other hallucinations (olfactory, gustatory and tactile).


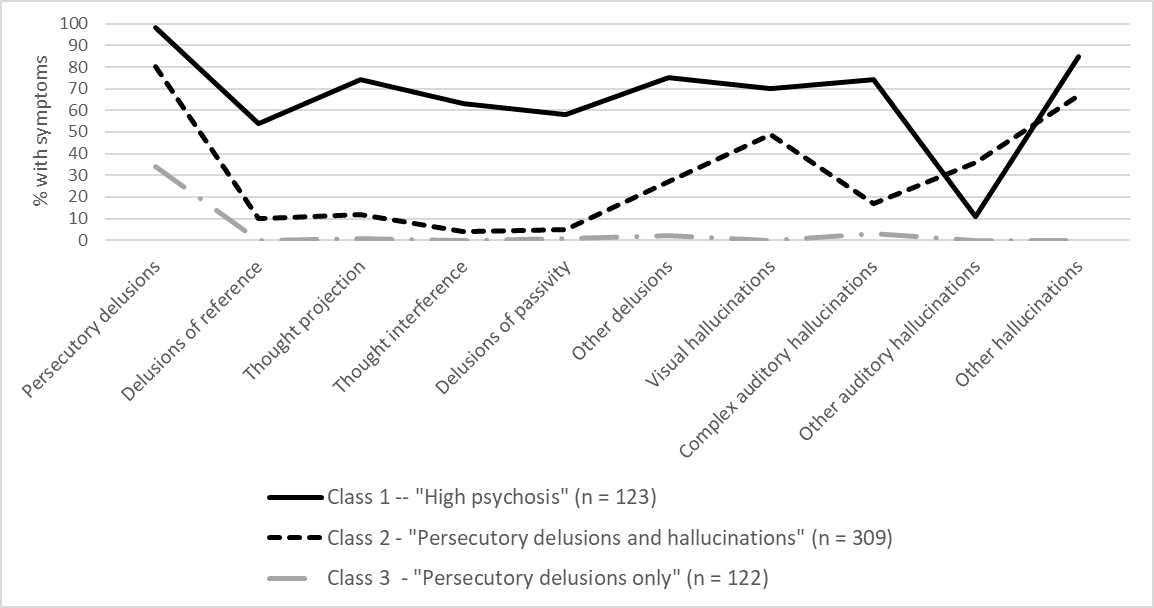


**Figure S1**. Lifetime symptom prevalence for the three-class models for the full sample.


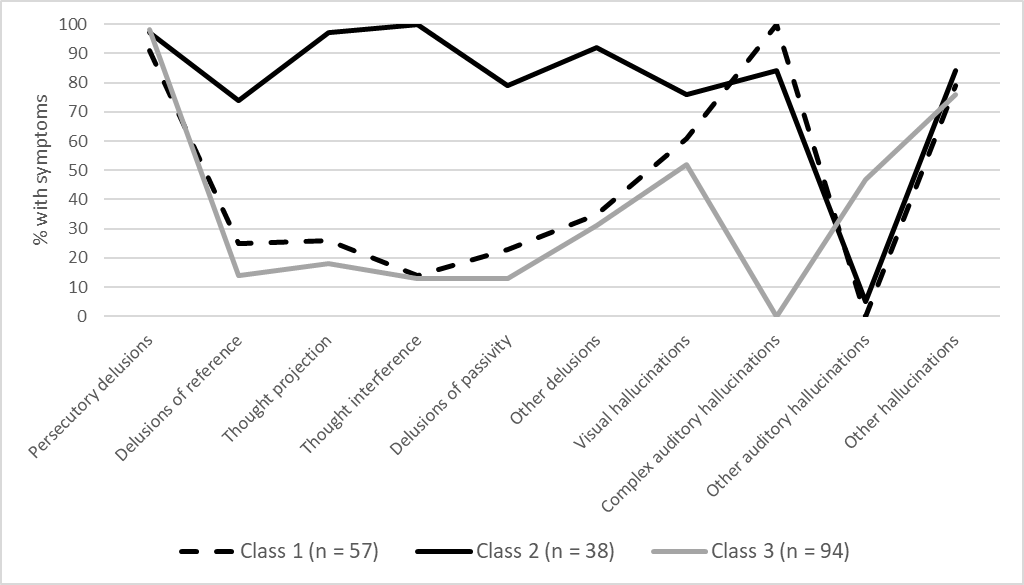


**Figure S2**. Lifetime symptom prevalence for the three-class models for participants who met the symptom criteria for schizophrenia.

**Table S1.** Characteristics by class for three-class model in the full sample

|  | Class 1  (n = 123) | Class 2  (n = 309) | Class 3  (n = 122) | Total sample (N = 554) |
| --- | --- | --- | --- | --- |
| Symptoms (%) |  |  |  |  |
| Persecutory delusions | 98 | 80*** | 34***††† | 74 |
| Delusions of reference | 54 | 10*** | 0***††† | 18 |
| Thought projection | 74 | 12*** | 1***††† | 23 |
| Thought interference | 63 | 4*** | 0***††† | 16 |
| Delusions of passivity | 58 | 5*** | 1***††† | 16 |
| Other delusions | 75 | 27*** | 2***††† | 32 |
| Visual hallucinations | 70 | 49*** | 0***††† | 43 |
| Complex auditory hallucinations | 74 | 17*** | 3***††† | 27 |
| Other auditory hallucinations | 11 | 36*** | 0***††† | 23 |
| Other hallucinations^a^ | 85 | 67*** | 0***††† | 56 |
| Demographics |  |  |  |  |
| Age (median years) | 33 | 33 | 35 | 34 |
| Male (%) | 71 | 69 | 75 | 71 |
| Years of schooling (median) | 10 | 10 | 10 | 10 |
| Unemployed (%) | 82 | 75 | 73 | 76 |
| Immigrant (%) | 17 | 8** | 12 | 11 |
| Methamphetamine use |  |  |  |  |
| Duration of use (median years) | 15 | 14 | 15 | 14 |
| Days of use (median) | 14 | 15 | 13 | 14 |
| Injecting (%) | 80 | 71 | 71 | 73 |
| SDS score (%) | 9 | 8 | 7 | 8 |
| Dependent (%) | 81 | 79 | 74 | 78 |
| No. other drug classes used in past month) (mean) | 3.3 | 3.4 | 3.1† | 3 |
| DSM-IV criteria for schizophrenia (%) | 26 | 3*** | 0*** | 7 |
| Meets symptom criteria for schizophrenia (%) | 63 | 35*** | 3***††† | 66 |

* p < .05, ** p < .01, ***p < .001, relative to Class 1

† p < .05, †† p < .01, †††p < .001, relative to Class 2

Tactile, gustatory or olfactory
